# Relationship between diet quality and nonalcoholic fatty liver disease predictor indices in Iranian patients with metabolic syndrome: A cross‐sectional study

**DOI:** 10.1002/fsn3.3549

**Published:** 2023-07-04

**Authors:** Maryam Taghdir, Akram Salehi, Karim Parastouei, Sepideh Abbaszadeh

**Affiliations:** ^1^ Health Research Centre, Life Style Institute Baqiyatallah University of Medical Sciences Tehran Iran; ^2^ Department of Nutrition and Food Hygiene, Faculty of Health Baqiyatallah University of Medical Sciences Tehran Iran; ^3^ Student Research Committee Baqiyatallah University of Medical Sciences Tehran Iran

**Keywords:** Fatty Liver Index, Healthy Eating Index‐2015, Hepatic Steatosis Index, metabolic syndrome, Triglyceride‐Glucose Index

## Abstract

The present study aimed to assess the association between diet quality and nonalcoholic fatty liver disease (NAFLD) predictor indices in patients with metabolic syndrome (MetS). This cross‐sectional study was carried out among 344 adult patients with MetS. The diet quality of patients was calculated by Healthy Eating Index‐2015 (HEI‐2015). NAFLD predictor indices (Hepatic Steatosis Index [HSI], Triglyceride‐Glucose Index [TyG], and Fatty Liver Index [FLI]) were calculated and compared according to the HEI‐2015 quartiles. The relationship between the HEI‐2015 score and HSI, FLI, and TyG Index was estimated using multiple linear regression analysis. The findings of the present study revealed that patients with the highest HEI score had the lowest FLI score (*p* = .003) and HSI score (*p* = .05). There was an inverse relationship between the HEI‐2015 score and FLI (*β* = −0.49; *p* < .001), HSI (*β* = −0.05; *p* = .25), and TyG Index (*β* = −0.002; *p* = .34). According to our result, after adjusting for possible confounding factors, there was a statistically significant inverse association between HEI‐2015 and FLI.

## Graphical abstract

The findings of the present study revealed that patients with the highest HEI score had the lowest FLI score and HSI score. According to our result, after adjusting for possible confounding factors, there was a statistically significant inverse association between HEI‐2015 and FLI.

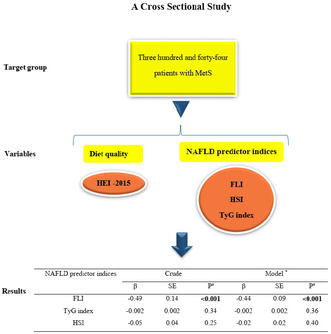

## 1 INTRODUCTION

A set of metabolic disorders including, increased fasting blood sugar (FBS), blood pressure (BP), triglycerides, and decreased high‐density lipoprotein cholesterol (HDL‐C) along with abdominal obesity constitute the metabolic syndrome (MetS) (Eckel et al., 2005). The prevalence of MetS is increasing globally (Tabatabaei‐Malazy et al., 2021). The overall prevalence of MetS in Iran (based on the criteria of the International Diabetes Federation [IDF]) is estimated at 43.5%. It is also more common in women and urban residents than in men and rural populations (Tabatabaei‐Malazy et al., 2021). MetS is usually accompanied by cardiovascular disease (CVD), diabetes mellitus (type 2), and nonalcoholic fatty liver disease (NAFLD) (Eslam et al., 2020; Katsiki et al., 2018). Recently, it has been suggested that metabolic‐associated fatty liver disease (MAFLD) is a more suitable term for NAFLD associated with metabolic disorders (Eslam et al., 2020). NAFLD may lead to steatohepatitis, fibrosis, and cirrhosis (Matteoni et al., 1999). The gold standard for diagnosing NAFLD is liver biopsy, which is an invasive procedure and has adverse consequences for the patient (Sumida et al., 2014). In recent years, some indices such as Hepatic Steatosis Index (HSI), Fatty Liver Index (FLI), and Triglyceride‐Glucose Index (TyG) have been considered as noninvasive methods to predict liver fat changes (Khamseh et al., 2021; Lee et al., 2010; Motamed et al., 2016).

Lifestyle modifications, including physical activity and adherence to a balanced diet, are recommended to prevent and improve NAFLD (Liver & Diabetes, 2016). Some studies have shown that there is a relationship between diet quality and NAFLD (Chan et al., 2015; Doustmohammadian et al., 2022; Vahid et al., 2019). Diet quality is evaluated using several indices. According to the 2015–2020 Dietary Guidelines for Americans, the Healthy Eating Index‐2015 (HEI‐2015) is a measure for assessing the extent of adherence to dietary recommendations. HEI‐2015 evaluates components such as whole fruits, vegetables, whole grains, plant proteins, and fatty acids (Reedy et al., 2018). A negative correlation between the HEI‐2015 score and CVD and all‐cause mortality has been reported (Hu et al., 2020). To our knowledge, this is the first study on the association between diet quality and NAFLD predictor indices (FLI, HSI, and TyG Index) in patients with MetS.

## 2 MATERIALS AND METHODS

### 2.1 Study population

In this cross‐sectional study, a total of 344 patients who which randomly selected from patients with MetS who were referred to a referral clinic (Amiralmomenin clinic) in Mobarakeh, Isfahan province, Iran, between July 2021 and March 2022 were studied.

MetS was diagnosed based on the IDF criteria (a waist circumference (WC) ≥94 cm in men and ≥80 cm in women and having two of the following five criteria: systolic BP: ≥130 mm/Hg, diastolic BP: ≥85 mm/Hg, FBS: ≥100 mg/dL, triglyceride: ≥150 mg/dL, and HDL‐C: <50 mg/dL in women and <40 mg/dL in men) (Alberti et al., 2005). Exclusion criteria were having cancer or a history of chronic liver, kidney, and thyroid diseases, alcohol consumption, and being pregnant or breastfeeding.

Written informed consent was obtained from all participants. The Ethics Committee for Research at Baqiyatallah University of Medical Sciences has approved the protocol of study (IR.BMSU.REC.1400.058).

### 2.2 Anthropometric and blood pressure measurements

Weight was measured via the Seca scale (Seca) with an accuracy of 100 g, and height was measured, with a precision of 0.5 cm, using the Seca stadiometer (Seca). The BMI was calculated using the formula. WC was measured in the narrowest area between the last rib and the iliac crest with a nonelastic tape measure with an accuracy of 0.1 cm.

BP was measured while sitting and using a digital BP monitor, three times with an interval of at least 5 min, and their average was recorded.

### 2.3 Laboratory tests

Blood samples, after 12 h of fasting, were taken from all patients, and biochemical parameters were measured. FBS, triglyceride, HDL‐C, alanine aminotransferase (ALT), aspartate aminotransferase (AST), and gamma‐glutamyl‐transferase (GGT) were measured using Pars Azmoon kit (Pars Azmoon Co.) and autoanalyzer BS200 (Mindray).

### 2.4 Measurement of NAFLD predictor indices

NAFLD predictor indices were calculated through the following formulas:

FLI = (e^0.953×Ln (triglycerides)+0.139×BMI+0.718×Ln (GGT)+0.053×WC−15.745^)/(1 + e^0.953×Ln (triglycerides)+0.139×BMI+0.718×Ln (GGT)+0.053×WC−15.745^) × 100 (Bedogni et al., 2006)

TyG Index = Ln (triglycerides [mg/dl] × glucose [mg/dl]/2) (Simental‐Mendía et al., 2008).

HSI = 8 × (ALT/AST) + BMI + 2 (if type 2 diabetes) + 2 (if female) (Fennoun et al., 2020).

The classification of NAFLD predictor indices in terms of the risk of developing NAFLD is as follows:

FLI: <30= (Low risk), 30< to < 60= Indeterminate risk and ≥60= High risk

HSI: < 30= No risk, 30< to <36= Indeterminate Risk, ≥36= High Risk

TyG Index: optimal cut‐off value to predict NAFLD is approximately 8.5 (Correa et al., 2021).

### 2.5 Diet assessment

Patients' food intake was estimated via a valid semiquantitative food frequency questionnaire (FFQ) (147 food items) (Mirmiran et al., 2010). A trained nutritionist determined the frequency of consumption (daily, weekly, monthly, and yearly) of each food item through an interview. Macro‐ and micronutrients intake, as well as energy intake was calculated based on the modified nutritionist IV for Iranian foods (First Databank Inc.).

HEI‐2015 consists of 13 components (Krebs‐Smith et al., 2018): adequacy components that include (total and whole fruits, total vegetables, greens and beans, total protein foods, whole grains, dairy, seafood and plant proteins, and fatty acids) (polyunsaturated fatty acids [PUFAs] + monounsaturated fatty acids [MUFAs]/saturated fatty acids [SFAs]), and moderation components that include (saturated fats, sodium, added sugars, and refined grains). Added sugars and saturated fats were converted to the percentage of total energy intake, and other components except fatty acids were converted to cups/oz or grams per 1000 kcal intake.

Total fruits, total vegetables, whole fruits, total protein foods, greens and beans, and seafood and plant proteins scored 0 (lowest) and 5 (highest) based on daily intake. The score of daily consumption of fatty acids, whole grains, and dairy was between 0 (lowest) and 10 (highest). Refined grains, added sugars, sodium, and saturated fats scored 0 (highest) and 10 (lowest) based on daily intake. Therefore, the HEI‐2015 total score ranges between 0 and 100.

### 2.6 Statistical analysis

Data were shown as mean ± standard deviation (SD) and percentages (*n* [%]) for continuous and categorical variables, respectively. The total HEI‐2015 was classified into quartiles according to their HEI‐2015 score. One‐way analysis of variance (ANOVA) was used to compare the average of the continuous variables across quartiles. A chi‐squared test was used to compare the frequencies between categorical variables. Simple and multiple linear regression were used to assess the adjusted effects of variables affecting the NAFLD predictor indices. In the adjusted model, the following variables were entered as covariates to control for confounding: age, sex, BMI, calorie, carbohydrate, and fat intake. A *p* value <.05 was considered statistically significant. All analyses were performed using SPSS software version 22 (SPSS Inc.).

## 3 RESULTS

The total number of participants was 344. There was 92 (26.5%) males. The mean age and BMI of the participants were 61.86 ± 10.21 years and 32.16 ± 4.22 kg/m^2^, respectively. The patients were divided into quartiles according to their HEI‐2015 scores as follows: Quartile 1: 50–69.25; Quartile 2: 69.26–73.67; Quartile 3: 73.68–77.49; and Quartile 4: ≥77.5. Table 1 shows the patient's characteristics. Patients in the highest quartile of the HEI‐2015 score were younger (*p* = .04); and had lower BMI (*p* = .12) and WC (*p* = .29) compared with those in the lowest quartile. Regarding the NAFLD predictor indices, there were differences in FLI and HSI scores across the HEI‐2015 quartiles, and patients with the highest HEI score had the lowest FLI (*p* = .003) and HSI scores (*p* = .05).

**TABLE 1:** The characteristics of patients with metabolic syndrome by the lowest (1) and highest (4) quartiles of HEI‐2015 score.

Characteristics	Quartile 1 (*N* = 86)	Quartile 2 (*N* = 86)	Quartile 3 (*N* = 86)	Quartile 4 (*N* = 86)	*p* value*
Age, years	63.51 ± 9.34	61.83 ± 9.81	62.79 ± 11.44	59.36 ± 9.88	**.04**
Sex (male)	23 (26.7)	21 (24.4)	28 (32.6)	20 (23.3)	.52
Marital status (married)	85 (98.8)	84 (97.7)	86 (100.0)	86 (100.0)	.29
Educational status					
Illiterate	18 (20.9)	25 (29.1)	24 (27.9)	20 (23.3)	
Diploma or less	65 (75.6)	54 (62.8)	58 (67.5)	60 (69.7)	.64
Bachelor of science	3 (3.5)	5 (5.8)	4 (4.6)	5 (5.8)	
Postgraduate	0 (0)	2 (2.3)	0 (0)	1 (1.2)	
BMI (kg/m^2^)	32.4 ± 42.40	31.4 ± 87.15	32.04 ± 93.52	31.03 ± 47.69	.12
WC (cm)	104.8 ± 27.56	104.9 ± 10.29	104.10 ± 63.20	102.9 ± 11.33	.29
FLI	76.2 ± 12.48	69.80 ± 19.35	72.30 ± 18.18	66.71 ± 17.51	**.003**
TyG Index	4.85 ± 0.31	4.83 ± 0.28	4.80 ± 0.27	4.81 ± 0.26	.63
HSI	43.11 ± 5.14	43.00 ± 5.70	43.97 ± 6.06	41.69 ± 4.79	.05

Significant results are shown in bold.

*Note*: Values are provided as mean ± SD or *n* (%). Quartile 1: 50–69.25; Quartile 2: 69.26–73.67; Quartile 3: 73.68–77.49; and Quartile 4: ≥ 77.5.

Abbreviations: BMI, body mass index; FLI, Fatty Liver Index; HSI, Hepatic Steatosis Index; TyG Index, Triglyceride‐Glucose Index; WC, waist circumference.

* Statistical significance was set at the level of *p* < .05.

Table 2 displays the distribution of dietary intakes of macro‐ and micronutrients of participants across the quartiles of the HEI‐2015 scores. According to Table 2, patients in the highest quartile of the HEI‐2015 score intake of more protein, vitamin D, vitamin E, vitamin C, vitamin B_6_, vitamin B_12_, calcium, and zinc compared with those in the lowest quartile.

**TABLE 2:** The distribution of dietary intakes of macro‐ and micronutrients of participants by the lowest (1) and highest (4) quartiles of HEI‐2015 score.

Characteristics (mean ± SD)	Quartile 1 (*N* = 86)	Quartile 2 (*N* = 86)	Quartile 3 (*N* = 86)	Quartile 4 (*N* = 86)	*p* value*
HEI‐2015 score	64.14 ± 4.24	71.60 ± 1.28	75.70 ± 1.21	80.75 ± 2.78	
Energy intake (kcal/days)	2775.03 ± 920.75	2816.24 ± 723.06	3037.9 ± 928.85	2858.18 ± 943.19	.22
Total protein intake (g/days)	95.99 ± 39.18	105.79 ± 33.97	121.92 ± 50.68	121.18 ± 43.58	**<.001**
Total carbohydrate intake (g/days)	391.84 ± 140.87	396.60 ± 115.47	407.96 ± 120.65	377.90 ± 132.58	.48
Total fat intake (g/days)	97.30 ± 45.19	96.63 ± 36.11	109.90 ± 42.48	104.92 ± 42.91	.11
Vitamin D (μg/days)	1.53 ± 1.65	2.47 ± 2.76	2.84 ± 2.04	3.06 ± 3.03	**<.001**
Vitamin E (mg/days)	14.40 ± 9.44	13.26 ± 6.01	17.03 ± 8.94	19.43 ± 10.97	**<.001**
Vitamin C (mg/days)	144.25 ± 93.50	189.62 ± 112.02	223.62 ± 108.09	245.94 ± 117.97	**<.001**
Vitamin B_6_ (mg/days)	2.14 ± 0.82	2.46 ± 0.73	2.79 ± 1.08	2.85 ± 1.04	**<.001**
Vitamin B_9_ (μg/d)	574.39 ± 220.46	609.35 ± 185.54	644.81 ± 244.11	644.52 ± 221.37	.10
Vitamin B_12_ (μg/days)	5.69 ± 3.01	6.80 ± 3.35	7.55 ± 4.26	7.18 ± 3.40	**.005**
Zinc (mg/days)	14.74 ± 5.86	16.54 ± 5.01	18.71 ± 8.19	18.32 ± 7.15	**<.001**
Calcium (mg /days)	1342.03 ± 697.34	1616.724 ± 706.40	1736.71 ± 772.36	1739.58 ± 717.66	**.001**

Significant results are shown in bold.

*Note*: Quartile 1: 50–69.25; Quartile 2: 69.26–73.67; Quartile 3: 73.68–77.49; and Quartile 4: ≥77.5.

Abbreviation: HEI‐2015, Healthy Eating Index‐2015.

* Statistical significance was set at the level of *p* < .05.

Patients in the highest quartile of the HEI‐2015 score intake more total fruits (*p* < .001), whole fruits (*p* < .001), total vegetables (*p* < .001), greens and beans (*p* < .001), dairy (*p* < .001), total protein foods (*p* < .001), seafood and plant proteins (*p* < .001), and healthy fats (*p* < .001) as well as less refined grains (*p* = .006) than patients in the lowest quartile (not shown in the table).

Table 3 indicates the relationship between NAFLD predictor indices and HEI‐2015. According to the crude and adjusted models, there was an inverse relationship between the HEI‐2015 and FLI (*β* = −0.49; *p* < .001 and *β* = −0.44; *p* < .001, respectively), HSI (*β* = −0.05; *p* = .25 and β = −0.02; *p* = .40, respectively), and TyG Index (*β* = −0.002; *p* = .34 and *β* = −0.002; *p* = .36, respectively).

**TABLE 3:** The crude and adjusted model for the association between HEI‐2015 and NAFLD predictor indices in patients with MetS.

NAFLD predictor indices	Crude			Model^a^		
	*β*	SE	*p* *	*β*	SE	*p* *
FLI	−0.49	0.14	**<.001**	−0.44	0.09	**<.001**
TyG Index	−0.002	0.002	.34	−0.002	0.002	.36
HSI	−0.05	0.04	.25	−0.02	0.02	.40

Significant results are shown in bold.

Abbreviations: FLI, Fatty Liver Index; HSI, Hepatic Steatosis Index, TyG Index, Triglyceride‐Glucose Index.

^a^ Adjusted for age, sex, energy, carbohydrate and fat intake, and BMI.

* Statistical significance was set at the level of *p* < .05.

## 4 DISCUSSION

NAFLD, as a serious complication of MetS, is a main risk factor for chronic liver diseases (Eslam et al., 2020; Matteoni et al., 1999). Various studies have shown that FLI, HSI, and TyG Index are accurate, simple, and valuable tools to predict and diagnose NAFLD (Huang et al., 2015; Khamseh et al., 2021; Sourianarayanane & McCullough, 2022). Identifying the factors influencing the occurrence of NAFLD plays an essential role in preventing disease. The most recommended treatment for NAFLD is lifestyle modification with an emphasis on both physical activity and a healthy diet (Liver & Diabetes, 2016). HEI‐2015 is a well‐known measure of diet quality (Krebs‐Smith et al., 2018). We evaluate the relationship between the HEI‐2015 score and NAFLD predictor indices (FLI, HSI, and TyG Index) in patients with MetS.

Our results showed that there was a negative association between HEI‐2015 and FLI, HSI, and TyG Index; however, it was statistically significant only between HEI‐2015 and FLI. Similar to the finding of the present study, the results of another study showed that the HEI‐2015 score in adults with a higher FLI score was significantly poorer compared with adults with a lower FLI score (Madril et al., 2021). Various studies have shown that there was a negative association between diet quality and NAFLD or fibrosis risk (Kalafati et al., 2019; Ma et al., 2018; Soleimani et al., 2019). A cohort study revealed that a higher HEI‐2015 score was related to a lower risk of NAFLD (Park et al., 2020). In a study with 3589 participants in the United States, a lower risk of NAFLD was reported to be associated with a higher HEI score (Vilar‐Gomez et al., 2022). In another study, a healthy diet (higher HEI‐2015 score) was related to lower all‐cause and cardiovascular mortality in patients with NAFLD (Vilar‐Gomez et al., 2023).

A higher HEI‐2015 score, which indicates a high‐quality diet, shows more intake of vegetables, fruits, seafood, and plant proteins, whole grains, healthier fatty acids, and a lower intake of refined grains, sodium, and added sugars (Krebs‐Smith et al., 2018). In the current study, patients who were in the highest HEI‐2015 quartile consumed more fruits, dairy, vegetables, greens and beans, seafood and plant proteins, and healthy fatty acids, as well as fewer refined grains than patients in the lowest quartile. Furthermore, compared with patients in the first HEI‐2015 quartile, FLI and HSI scores were lower in patients in the highest quartile.

HEI‐2015 components could be related to the reduction of NAFLD risk by different mechanisms. Considering that obesity is one of the causes of NAFLD (Rich et al., 2018), consuming fiber, from whole grains, vegetables, and fruits, by inducing satiety could contribute to weight loss (Nöthlings et al., 2008). In addition, the fermentation process of dietary fibers by intestinal bacteria leads to the production of short‐chain fatty acids (SCFAs). SCFAs may prevent NAFLD by regulating inflammation, appetite, fatty acid oxidation, and insulin resistance (Zhang et al., 2021). Furthermore, oxidative stress is involved in the initiation and progression of NAFLD (Rives et al., 2020), and therefore, vitamins, polyphenols, phytochemicals, and carotenoids in fruits and vegetables could prevent NAFLD due to their antioxidant properties (Ahmadi et al., 2023; Saini et al., 2015; Salomone et al., 2016). A statistically significant inverse relationship between dietary carotenoid intake and the US FLI score was revealed by Christensen et al. (2019).

It has been shown that healthy fatty acids (MUFAs and PUFAs [particularly omega‐3 FAs]) could prevent NAFLD by reducing triglycerides, glucose, oxidative stress, and hepatic steatosis as well as improving insulin sensitivity, anti‐inflammatory effect, and inhibiting hepatic fat accumulation (Berge et al., 2014; Kamari et al., 2023; Spooner & Jump, 2019). The main sources of omega‐3 FAs are seafood, seeds, nuts, and plant derivatives (Calder, 2013). In a cross‐sectional study, nut consumption was associated with a lower risk of NAFLD (calculated by FLI) in older adults (Tan et al., 2021). In another study, it was reported that an omega‐3 PUFA‐rich diet (fish oil diet) reduced the risk of NAFLD by inhibiting acetyl‐CoA carboxylase and sterol regulatory element‐binding protein‐1c (SREBP‐1c) (Zhou et al., 2021). Khan and Jackson (2018) found a significant relationship between pro‐inflammatory omega‐6 FAs intake and FBS, triglyceride, and cholesterol. In this study, the main sources of omega‐6 FAs were red meat, chicken, and dairy products.

Several studies have shown that the Mediterranean diet, which is rich in whole grains, fruits and vegetables, monounsaturated fats, omega‐3 FAs, and plant proteins as well as low in saturated fat is related to lower risk and progression of NAFLD (Baratta et al., 2017; Kastorini & Panagiotakos, 2010; Saeed et al., 2019). On the other hand, it has been determined that Western dietary patterns which are characterized by a high intake of fast foods, refined grains, added sugar, sodium, and saturated fats are associated with high NAFLD risk due to increased insulin resistance, weight gain, oxidative stress, and pro‐inflammatory processes (Kalafati et al., 2019; Soleimani et al., 2019; Trovato et al., 2018). The result of a meta‐analysis showed that higher sodium intake is related to a higher risk of NAFLD (Shojaei‐Zarghani et al., 2022).

The current study has some strengths and limitations. The current research is the first study on the association between the HEI‐2015 score and NAFLD predictor indices (FLI, HSI, and TyG Index) in patients with MetS. According to the study design (cross‐sectional), it was not possible to assess the causality, so it is suggested to conduct prospective cohort studies. While validated FFQ was used, there is the possibility of recall bias. However, the collection of data from the questionnaire by a trained nutritionist reduced recall bias. Despite adjusting for important confounding factors, there may still be other confounders that affect the results of this study.

According to our results, in patients with MetS, HEI‐2015 score and NAFLD predictor indices (FLI, HSI, and TyG Index) were inversely correlated; however, this relationship was statistically significant only in the case of FLI. Adherence to a diet containing whole grains, fruits, vegetables, plant‐based protein foods, and healthy fats could be beneficial in preventing NAFLD in patients with MetS.

## AUTHOR CONTRIBUTIONS

**Maryam Taghdir:** Conceptualization (lead); data curation (equal); formal analysis (equal); investigation (equal); methodology (equal); project administration (lead); supervision (lead); validation (equal); visualization (equal); writing – original draft (lead); writing – review and editing (equal). **Akram Salehi:** Conceptualization (equal); data curation (equal); formal analysis (equal); investigation (equal); methodology (equal); writing – original draft (equal); writing – review and editing (equal). **Karim Parastouei:** Conceptualization (equal); data curation (equal); formal analysis (equal); investigation (equal); methodology (equal); project administration (equal); validation (equal); visualization (equal); writing – original draft (equal); writing – review and editing (equal). **Sepideh Abbaszadeh:** Conceptualization (equal); data curation (equal); formal analysis (equal); investigation (equal); methodology (equal); project administration (equal); validation (equal); visualization (equal); writing – original draft (equal); writing – review and editing (equal).

## FUNDING INFORMATION

This research did not receive any specific grant from funding agencies in the public, commercial, or not‐for‐profit sectors.

## CONFLICT OF INTEREST STATEMENT

No conflicts of interest to declare.

## ETHICS APPROVAL

The study was approved by the ethics committee for Research at Baqiyatallah University of Medical Sciences (IR.BMSU.REC.1400.058).

## Data Availability

The data that support the findings of this study are available from the corresponding author upon reasonable request.

## References

[fsn33549-bib-0001] Ahmadi, B. , Ramezani Ahmadi, A. , Jafari, M. , & Morshedzadeh, N. (2023). The association of dietary phytochemical index and nonalcoholic fatty liver disease. Food Science & Nutrition, 00, 1–10. 10.1002/fsn3.3389 PMC10345673 37457157

[fsn33549-bib-0002] Alberti, K. G. M. , Zimmet, P. , & Shaw, J. (2005). The metabolic syndrome—A new worldwide definition. The Lancet, 366, 1059–1062. 10.1016/S0140-6736(05)67402-8 16182882

[fsn33549-bib-0003] Baratta, F. , Pastori, D. , Polimeni, L. , Bucci, T. , Ceci, F. , Calabrese, C. , Ernesti, I. , Pannitteri, G. , Violi, F. , & Angelico, F. (2017). Adherence to mediterranean diet and non‐alcoholic fatty liver disease: Effect on insulin resistance. Official Journal of the American College of Gastroenterology, 112, 1832–1839. 10.1038/ajg.2017.371 29063908

[fsn33549-bib-0004] Bedogni, G. , Bellentani, S. , Miglioli, L. , Masutti, F. , Passalacqua, M. , Castiglione, A. , & Tiribelli, C. (2006). The fatty liver index: A simple and accurate predictor of hepatic steatosis in the general population. BMC Gastroenterology, 6, 1–7. 10.1186/1471-230X-6-33 17081293 PMC1636651

[fsn33549-bib-0005] Berge, K. , Musa‐Veloso, K. , Harwood, M. , Hoem, N. , & Burri, L. (2014). Krill oil supplementation lowers serum triglycerides without increasing low‐density lipoprotein cholesterol in adults with borderline high or high triglyceride levels. Nutrition Research, 34, 126–133. 10.1016/j.nutres.2013.12.003 24461313

[fsn33549-bib-0006] Calder, P. C. (2013). Omega‐3 polyunsaturated fatty acids and inflammatory processes: Nutrition or pharmacology? British Journal of Clinical Pharmacology, 75, 645–662. 10.1111/j.1365-2125.2012.04374.x 22765297 PMC3575932

[fsn33549-bib-0007] Chan, R. , Wong, V. W. S. , Chu, W. C. W. , Wong, G. L. H. , Li, L. S. , Leung, J. , Chim, A. M. L. , Yeung, D. K. W. , Sea, M. M. M. , & Woo, J. (2015). Diet‐quality scores and prevalence of nonalcoholic fatty liver disease: A population study using proton‐magnetic resonance spectroscopy. PLoS One, 10, e0139310. 10.1371/journal.pone.0139310 26418083 PMC4587971

[fsn33549-bib-0008] Christensen, K. , Lawler, T. , & Mares, J. (2019). Dietary carotenoids and non‐alcoholic fatty liver disease among US adults, NHANES 2003–2014. Nutrients, 11, 1101. 10.3390/nu11051101 31108934 PMC6566688

[fsn33549-bib-0009] Correa, T. L. , Guelli, M. S. T. C. , & De Oliveira, I. O. (2021). Triglyceride‐glucose index (TyG) is positively associated with nonalcoholic fatty liver disease. American Heart Journal, 242, 158–159. 10.1016/j.ahj.2021.10.035

[fsn33549-bib-0010] Doustmohammadian, A. , Clark, C. C. , Maadi, M. , Motamed, N. , Sobhrakhshankhah, E. , Ajdarkosh, H. , Mansourian, M. R. , Esfandyari, S. , Hanjani, N. A. , & Nikkhoo, M. (2022). Favorable association between Mediterranean diet (MeD) and DASH with NAFLD among Iranian adults of the Amol Cohort Study. Scientific Reports, 12, 2131. 10.1038/s41598-022-06035-8 35136128 PMC8825797

[fsn33549-bib-0011] Eckel, R. H. , Grundy, S. M. , & Zimmet, P. Z. (2005). The metabolic syndrome. The Lancet, 365, 1415–1428. 10.1016/S0140-6736(05)66378-7 15836891

[fsn33549-bib-0012] Eslam, M. , Sanyal, A. J. , George, J. , Sanyal, A. , Neuschwander‐Tetri, B. , Tiribelli, C. , Kleiner, D. E. , Brunt, E. , Bugianesi, E. , & Yki‐Järvinen, H. (2020). MAFLD: A consensus‐driven proposed nomenclature for metabolic associated fatty liver disease. Gastroenterology, 158(1999–2014), 1999–2014.e1. 10.1053/j.gastro.2019.11.312 32044314

[fsn33549-bib-0013] European Association for the Study of the Liver (EASL) , European Association for the Study of Diabetes (EASD) , & European Association for the Study of Obesity (EASO) . (2016). EASL‐EASD‐EASO Clinical Practice Guidelines for the management of non‐alcoholic fatty liver disease. Obesity Facts, 9, 65–90. 10.1159/000443344 27055256 PMC5644799

[fsn33549-bib-0014] Fennoun, H. , El Mansouri, S. , Tahiri, M. , Haraj, N. E. , El Aziz, S. , Hadad, F. , Hliwa, W. , Badr, W. , & Chadli, A. (2020). Interest of hepatic steatosis index (HSI) in screening for metabolic steatopathy in patients with type 2 diabetes. The Pan African Medical Journal, 37, 1–7. 10.11604/pamj.2020.37.270.9087 PMC7864273 33598084

[fsn33549-bib-0015] Hu, E. A. , Steffen, L. M. , Coresh, J. , Appel, L. J. , & Rebholz, C. M. (2020). Adherence to the healthy eating index–2015 and other dietary patterns may reduce risk of cardiovascular disease, cardiovascular mortality, and all‐cause mortality. The Journal of Nutrition, 150, 312–321. 10.1093/jn/nxz218 31529069 PMC7373820

[fsn33549-bib-0016] Huang, X. , Xu, M. , Chen, Y. , Peng, K. , Huang, Y. , Wang, P. , Ding, L. , Lin, L. , Xu, Y. , & Chen, Y. (2015). Validation of the fatty liver index for nonalcoholic fatty liver disease in middle‐aged and elderly Chinese. Medicine, 94, e1682. 10.1097/MD.0000000000001682 26448014 PMC4616754

[fsn33549-bib-0017] Kalafati, I.‐P. , Borsa, D. , Dimitriou, M. , Revenas, K. , Kokkinos, A. , & Dedoussis, G. V. (2019). Dietary patterns and non‐alcoholic fatty liver disease in a Greek case–control study. Nutrition, 61, 105–110. doi:10.1016/j.nut.2018.10.032 30708259

[fsn33549-bib-0018] Kamari, N. , Moradinazar, M. , Qasemi, M. , Khosravy, T. , Samadi, M. , & Abdolahzad, H. (2023). Combination of the effect of ginger and anti‐inflammatory diet on children with obesity with nonalcoholic fatty liver disease: A randomized clinical trial. Food Science & Nutrition, 11, 1846–1859. 10.1002/fsn3.321 37051346 PMC10084988

[fsn33549-bib-0019] Kastorini, C.‐M. , & Panagiotakos, D. B. (2010). The role of the Mediterranean diet on the development of the metabolic syndrome. Frontiers in bioscience (Elite Edition), 2, 1320–1333. 10.2741/E192 20515804

[fsn33549-bib-0020] Katsiki, N. , Perez‐Martinez, P. , Anagnostis, P. , Mikhailidis, D. P. , & Karagiannis, A. (2018). Is nonalcoholic fatty liver disease indeed the hepatic manifestation of metabolic syndrome? Current Vascular Pharmacology, 16, 219–227. 10.2174/1570161115666170621075619 28669328

[fsn33549-bib-0021] Khamseh, M. E. , Malek, M. , Abbasi, R. , Taheri, H. , Lahouti, M. , & Alaei‐Shahmiri, F. (2021). Triglyceride glucose index and related parameters (triglyceride glucose‐body mass index and triglyceride glucose‐waist circumference) identify nonalcoholic fatty liver and liver fibrosis in individuals with overweight/obesity. Metabolic Syndrome and Related Disorders, 19, 167–173. 10.1089/met.2020.0109 33259744

[fsn33549-bib-0022] Khan, S. A. , & Jackson, R. T. (2018). Polyunsaturated fatty acids, inflammation, and metabolic syndrome in South Asian Americans in Maryland. Food Science & Nutrition, 6, 1575–1581. 10.1002/fsn3.698 30258600 PMC6145302

[fsn33549-bib-0023] Krebs‐Smith, S. M. , Pannucci, T. E. , Subar, A. F. , Kirkpatrick, S. I. , Lerman, J. L. , Tooze, J. A. , Wilson, M. M. , & Reedy, J. (2018). Update of the healthy eating index: HEI‐2015. Journal of the Academy of Nutrition and Dietetics, 118, 1591–1602. 10.1016/j.jand.2018.05.021 30146071 PMC6719291

[fsn33549-bib-0024] Lee, J. H. , Kim, D. , Kim, H. J. , Lee, C. H. , Yang, J. I. , Kim, W. , Kim, Y. J. , Yoon, J. H. , Cho, S. H. , & Sung, M. W. (2010). Hepatic steatosis index: A simple screening tool reflecting nonalcoholic fatty liver disease. Digestive and Liver Disease, 42, 503–508. 10.1016/j.dld.2009.08.002 19766548

[fsn33549-bib-0025] Ma, J. , Hennein, R. , Liu, C. , Long, M. T. , Hoffmann, U. , Jacques, P. F. , Lichtenstein, A. H. , Hu, F. B. , & Levy, D. (2018). Improved diet quality associates with reduction in liver fat, particularly in individuals with high genetic risk scores for nonalcoholic fatty liver disease. Gastroenterology, 155, 107–117. 10.1053/j.gastro.2018.03.038 29604292 PMC6035111

[fsn33549-bib-0026] Madril, P. , Taylor, C. , & Roberts, K. (2021). Differences in dietary intakes and overall diet quality by risk for non‐alcoholic fatty liver disease in US adults. Proceedings of the Nutrition Society, 80(OCE1), E40. 10.1017/S0029665121000410

[fsn33549-bib-0027] Matteoni, C. A. , Younossi, Z. M. , Gramlich, T. , Boparai, N. , Liu, Y. C. , & Mccullough, A. J. (1999). Nonalcoholic fatty liver disease: A spectrum of clinical and pathological severity. Gastroenterology, 116, 1413–1419. 10.1016/S0016-5085(99)70506-8 10348825

[fsn33549-bib-0028] Mirmiran, P. , Esfahani, F. H. , Mehrabi, Y. , Hedayati, M. , & Azizi, F. (2010). Reliability and relative validity of an FFQ for nutrients in the Tehran lipid and glucose study. Public Health Nutrition, 13, 654–662. 10.1017/S1368980009991698 19807937

[fsn33549-bib-0029] Motamed, N. , Sohrabi, M. , Ajdarkosh, H. , Hemmasi, G. , Maadi, M. , Sayeedian, F. S. , Pirzad, R. , Abedi, K. , Aghapour, S. , & Fallahnezhad, M. (2016). Fatty liver index vs waist circumference for predicting non‐alcoholic fatty liver disease. World Journal of Gastroenterology, 22, 3023–3030. 10.3748/wjg.v22.i10.3023 26973398 PMC4779925

[fsn33549-bib-0030] NöThlings, U. , Schulze, M. B. , Weikert, C. , Boeing, H. , Van Der Schouw, Y. T. , Bamia, C. , Benetou, V. , Lagiou, P. , Krogh, V. , & Beulens, J. W. (2008). Intake of vegetables, legumes, and fruit, and risk for all‐cause, cardiovascular, and cancer mortality in a European diabetic population. The Journal of Nutrition, 138, 775–781. 10.1093/jn/138.4.775 18356334

[fsn33549-bib-0031] Park, S.‐Y. , Noureddin, M. , Boushey, C. , Wilkens, L. R. , & Setiawan, V. W. (2020). Diet quality association with nonalcoholic fatty liver disease by cirrhosis status: The multiethnic cohort. Current Developments in Nutrition, 4, nzaa024. 10.1093/cdn/nzaa024 32190810 PMC7066377

[fsn33549-bib-0032] Reedy, J. , Lerman, J. L. , Krebs‐Smith, S. M. , Kirkpatrick, S. I. , Pannucci, T. E. , Wilson, M. M. , Subar, A. F. , Kahle, L. L. , & Tooze, J. A. (2018). Evaluation of the healthy eating index‐2015. Journal of the Academy of Nutrition and Dietetics, 118, 1622–1633. 10.1016/j.jand.2018.05.019 30146073 PMC6718954

[fsn33549-bib-0033] Rich, N. E. , Oji, S. , Mufti, A. R. , Browning, J. D. , Parikh, N. D. , Odewole, M. , Mayo, H. , & Singal, A. G. (2018). Racial and ethnic disparities in nonalcoholic fatty liver disease prevalence, severity, and outcomes in the United States: A systematic review and meta‐analysis. Clinical Gastroenterology and Hepatology, 16(198–210), 198–210.e2. 10.1016/j.cgh.2017.09.041 28970148 PMC5794571

[fsn33549-bib-0034] Rives, C. , Fougerat, A. , Ellero‐Simatos, S. , Loiseau, N. , Guillou, H. , Gamet‐Payrastre, L. , & Wahli, W. (2020). Oxidative stress in NAFLD: Role of nutrients and food contaminants. Biomolecules, 10, 1702. 10.3390/biom10121702 33371482 PMC7767499

[fsn33549-bib-0035] Saeed, N. , Nadeau, B. , Shannon, C. , & Tincopa, M. (2019). Evaluation of dietary approaches for the treatment of non‐alcoholic fatty liver disease: A systematic review. Nutrients, 11, 3064. 10.3390/nu11123064 31888132 PMC6950283

[fsn33549-bib-0036] Saini, R. K. , Nile, S. H. , & Park, S. W. (2015). Carotenoids from fruits and vegetables: Chemistry, analysis, occurrence, bioavailability and biological activities. Food Research International, 76, 735–750. 10.1016/j.foodres.2015.07.047 28455059

[fsn33549-bib-0037] Salomone, F. , Godos, J. , & Zelber‐Sagi, S. (2016). Natural antioxidants for non‐alcoholic fatty liver disease: Molecular targets and clinical perspectives. Liver International, 36, 5–20. 10.1111/liv.12975 26436447

[fsn33549-bib-0038] Shojaei‐Zarghani, S. , Safarpour, A. R. , Fattahi, M. R. , & Keshtkar, A. (2022). Sodium in relation with nonalcoholic fatty liver disease: A systematic review and meta‐analysis of observational studies. Food Science & Nutrition, 10, 1579–1591. 10.1002/fsn3.2781 35592291 PMC9094449

[fsn33549-bib-0039] Simental‐Mendía, L. E. , Rodríguez‐Morán, M. , & Guerrero‐Romero, F. (2008). The product of fasting glucose and triglycerides as surrogate for identifying insulin resistance in apparently healthy subjects. Metabolic Syndrome and Related Disorders, 6, 299–304. 10.1089/met.2008.0034 19067533

[fsn33549-bib-0040] Soleimani, D. , Ranjbar, G. , Rezvani, R. , Goshayeshi, L. , Razmpour, F. , & Nematy, M. (2019). Dietary patterns in relation to hepatic fibrosis among patients with nonalcoholic fatty liver disease. Diabetes, Metabolic Syndrome and Obesity: Targets and Therapy, 12, 315–324. 10.2147/DMSO.S198744 30881075 PMC6420105

[fsn33549-bib-0041] Sourianarayanane, A. , & Mccullough, A. J. (2022). Accuracy of steatosis and fibrosis NAFLD scores in relation to vibration controlled transient elastography: An NHANES analysis. Clinics and Research in Hepatology and Gastroenterology, 46, 101997. 10.1016/j.clinre.2022.101997 35842111

[fsn33549-bib-0042] Spooner, M. H. , & Jump, D. B. (2019). Omega‐3 fatty acids and nonalcoholic fatty liver disease in adults and children: Where do we stand? Current Opinion in Clinical Nutrition and Metabolic Care, 22, 103–110. 10.1097/MCO.0000000000000539 30601174 PMC6355343

[fsn33549-bib-0043] Sumida, Y. , Nakajima, A. , & Itoh, Y. (2014). Limitations of liver biopsy and non‐invasive diagnostic tests for the diagnosis of nonalcoholic fatty liver disease/nonalcoholic steatohepatitis. World Journal of Gastroenterology: WJG, 20, 475–485. 10.3748/wjg.v20.i2.475 24574716 PMC3923022

[fsn33549-bib-0044] Tabatabaei‐Malazy, O. , Saeedi Moghaddam, S. , Rezaei, N. , Sheidaei, A. , Hajipour, M. J. , Mahmoudi, N. , Mahmoudi, Z. , Dilmaghani‐Marand, A. , Rezaee, K. , & Sabooni, M. (2021). A nationwide study of metabolic syndrome prevalence in Iran; a comparative analysis of six definitions. PLoS One, 16, e0241926. 10.1371/journal.pone.0241926 33657130 PMC7928520

[fsn33549-bib-0045] Tan, S.‐Y. , Georgousopoulou, E. N. , Cardoso, B. R. , Daly, R. M. , & George, E. S. (2021). Associations between nut intake, cognitive function and non‐alcoholic fatty liver disease (NAFLD) in older adults in the United States: NHANES 2011–14. BMC Geriatrics, 21, 1–12. 10.1186/s12877-021-02239-1 34001034 PMC8127249

[fsn33549-bib-0046] Trovato, F. M. , Martines, G. F. , & Catalano, D. (2018). Addressing Western dietary pattern in obesity and NAFLD. Nutrire, 43, 1–6. 10.1186/s41110-018-0067-0

[fsn33549-bib-0047] Vahid, F. , Hekmatdoost, A. , Mirmajidi, S. , Doaei, S. , Rahmani, D. , & Faghfoori, Z. (2019). Association between index of nutritional quality and nonalcoholic fatty liver disease: The role of vitamin D and B group. The American Journal of the Medical Sciences, 358, 212–218. 10.1016/j.amjms.2019.06.008 31326093

[fsn33549-bib-0048] Vilar‐Gomez, E. , Nephew, L. D. , Vuppalanchi, R. , Gawrieh, S. , Mladenovic, A. , Pike, F. , Samala, N. , & Chalasani, N. (2022). High‐quality diet, physical activity, and college education are associated with low risk of NAFLD among the US population. Hepatology, 75, 1491–1506. 10.1002/hep.32207 34668597

[fsn33549-bib-0049] Vilar‐Gomez, E. , Vuppalanchi, R. , Gawrieh, S. , Pike, F. , Samala, N. , & Chalasani, N. (2023). Significant dose‐response association of physical activity and diet quality with mortality in adults with suspected NAFLD in a population study. The American Journal of Gastroenterology. 10.14309/ajg.0000000000002222 36799895

[fsn33549-bib-0050] Zhang, S. , Zhao, J. , Xie, F. , He, H. , Johnston, L. J. , Dai, X. , Wu, C. , & Ma, X. (2021). Dietary fiber‐derived short‐chain fatty acids: A potential therapeutic target to alleviate obesity‐related nonalcoholic fatty liver disease. Obesity Reviews, 22, e13316. 10.1111/obr.13316 34279051

[fsn33549-bib-0051] Zhou, N. , Du, S. , Dai, Y. , Yang, F. , & Li, X. (2021). ω3PUFAs improve hepatic steatosis in postnatal overfed rats and HepG2 cells by inhibiting acetyl‐CoA carboxylase. Food Science & Nutrition, 9, 5153–5165. 10.1002/fsn3.2482 34532024 PMC8441356

